# Physical Activity and Physical Fitness among University Students—A Systematic Review

**DOI:** 10.3390/ijerph19010158

**Published:** 2021-12-24

**Authors:** Vidran Kljajević, Mima Stanković, Dušan Đorđević, Drena Trkulja-Petković, Rade Jovanović, Kristian Plazibat, Mario Oršolić, Mijo Čurić, Goran Sporiš

**Affiliations:** 1 Secondary Vocational School, Bijelo Polje 84000, Montenegro; vidran.kljajevic@bijelopolje.co.me; 2 Faculty of Sport and Physical Education, University of Niš, 18000 Nis, Serbia; dusandjordjevic1995@gmail.com (D.Đ.); radejovanovic75@gmail.com (R.J.); 3 Faculty of Kinesiology, University of Zagreb, 10110 Zagreb, Croatia; drena.trkulja-petkovic@kif.unizg.hr (D.T.-P.); kristian.plazibat@gmail.com (K.P.); marioorsolic0@gmail.com (M.O.); goran.sporis@kif.unizg.hr (G.S.); 4 Faculty of Kinesiology, University of Osijek, 31000 Osijek, Croatia; mijo.curic@kifos.hr

**Keywords:** exercise, students, assessment, physical fitness, physical activity

## Abstract

The aim of this systematic review was to examine the scientific evidence regarding physical activity and physical fitness among university students. The search and analysis of the studies were done in accordance with the PRISMA guidelines. An electronic databases search (Google Scholar, PubMed, Science Direct, and Scopus) yielded 11,839 studies. Subsequently, the identified studies had to be published in English between 2011 and 2021, the experimental study had to have included males and females attending a faculty, and the participants had to have been evaluated for physical activity and fitness. A total of 21 studies were included in the quantitative synthesis, with a total of 7306 participants, both male and female. After analyzing the obtained results, it could be concluded that university students show a satisfactory level of physical activity and physical fitness. However, the results vary due to different factors involved, mostly related to the cultural differences and educational systems in different countries. As this study observes mediocre results of physical activity and physical fitness among university students, it is crucial to get their attention and awareness, to at least maintain a satisfactory level of physical activity and physical fitness.

## 1. Introduction

Diet and physical activity directly affect the health status of adults and children [1,2,3,4,5,6]. Due to the fact that the largest part of the world’s population is physically inactive, physical inactivity is considered to be a public health problem, as opposed to an individual problem. According to the report of the World Health Organization, physical inactivity is a risk factor, along with smoking, obesity, and hypertension [7]. Stress, obesity, and movement disorders such as hypokinesia are the most common causes of premature death, along with chronic non-communicable diseases from which neither children nor young people are immune [8]. Economically stable countries (about 60–70% of them) do not achieve even the minimum level of physical activity recommended by the World Health Organization in order to maintain health and energy balance [9,10].

Based on the abovementioned facts, the negative consequences of physical activity decline are also reflected in the student population, where the reduction of physical activity could also lead to decreased physical fitness. Health-related physical fitness is also influenced by many other factors, such as body weight and socioeconomic status. For example, the physical activity level of individuals of low socioeconomic status likely suffers due to their living areas providing less access to parks [11] and neighborhood walkability [12]. Additionally, their health is also negatively affected by the cost of healthy food compared to that of junk food [13]. Bodyweight disorder is very common in students and it can be often traced back to being overweight in childhood and adolescence [14]. Nevertheless, one of the most common negative external factors that influenced the exercise of physical activity in the students’ population is the lack of free time due to the schedule at the faculty, obligations in social and family life.

According to Caia et al. [15], low strength was peculiar to 61% of students and 28% had below-average strength. Kubieva et al. [16] concluded that students have problems with body mass index and strength, regardless of their physical activity level. Likewise, low cardiorespiratory fitness is also an important predictor and could be prevented only with lifestyle modifications [17], such as increasing physical activity and promoting dietary changes [18]. Kwan et al. [19] have already noticed the evident decline of physical activity when enrolling a university and according to several authors [20,21,22], already one-third of high school students are insufficiently active after transitioning to university life. This was confirmed by the study that investigated physical activity patterns among American, Asians, Africans and Hispanic university students. The authors have found that 46.7% of them didn’t engage in physical activity and 16.7% were physically inactive [23]. Several studies have also noted a weak physically active lifestyle and it is on the rise among university students [24,25,26].

Based on the above-mentioned facts, and due to the fact that many students feel pressure during the engagement in academic activities, with no time for physical activities, it was necessary to determine the current state of physical activity and physical fitness among university students. Therefore, the aim of this systematic review was to examine the scientific evidence regarding the level of physical activity and physical fitness among university students.

## 2. Materials and Methods

### 2.1. Literature Identification

Studies were searched and analyzed in accordance with the PRISMA (Preferred Reporting Items for Systematic Reviews and Meta-Analyses) guidelines [27]. In addition, this study is approved by the Ethical Board of Faculty of Sport and Physical Education, University of Niš. The research included studies conducted from 2011 to 2021 and the literature relevant for this type of research, available with the following databases: Google Scholar, PubMed, Science Direct, and Scopus.

The following keywords were used to search for the articles reporting on physical activity and physical fitness among university students: (“exercise” OR “physical activity” OR “accelerometer” OR “physical fitness” OR “strength” OR “endurance”) AND (“students” OR “adults” OR “students population” OR “university students” OR “college students”).

A descriptive method was used to analyze the data obtained, and all titles and abstracts were reviewed for possible study inclusion. At the same time, the identification strategy was modified and adapted to the particular database to increase the sensitivity. After a detailed identification process, studies were considered to be relevant if they met the inclusion criteria.

The search for studies, assessment of their value and data extraction were conducted independently by two authors (M.S. and D.Đ.), and the lists of references from previously assessed and original research were also reviewed. After that, each author cross-examined the identified studies, which were then taken for further analysis or rejected.

### 2.2. Inclusion Criteria

For the study to be included in the final analysis, it had to meet the following criteria: year of publication (2011 to 2021), the studies published in English, the experimental study included males and females attending faculty and that the participants were tested for the evaluation of physical activity and physical fitness.

### 2.3. Risk of Bias Assessment

The risk of bias was assessed according to the PRISMA guidelines, that is, using the PEDro scale [28] to determine the quality of reviewed studies and the potential risk of bias. Two independent authors (D.Ð. and M.S.) assessed the quality and risk of bias using checklists. Concordance between reviewers was estimated using k-statistics data to review the full text and assess relativity and risk of bias. In case of discordance as to findings of the risk of bias assessment, the obtained data was assessed by the third reviewer (K.P.), who also gave the final decision. The *k* rate of concordance between reviewers’ findings was *k* = 0.91.

### 2.4. Data Extraction

When the cross-examination was conducted, if the data were adequate, the necessary information was extracted and then moved to an Excel spreadsheet. The standardized data extraction protocol was applied (Cochrane Consumer and Communication Review Group’s) to extract the characteristics, such as authors and year of study, sample size, age, types of experimental program, duration, frequency, and study results.

## 3. Results

### 3.1. Quality of the Studies

Of the total number of studies that were included in the quantitative synthesis, and based on the points each study scored on the PEDro scale, the final study assessment scores were defined. According to Maher et al. [29], a score between 8–11 is considered to be optimal, but if the study gains between 0–3 points, that study will be classified with “poor” quality, 4–5 points with “fair” quality, 6–8 points with “good” quality, and 9–10 points with “excellent” quality. Of all studies included in this systematic review, 2 studies showed fair quality, 17 of them showed good quality, and the other 2 studies showed excellent quality, which is shown in Table 1.

### 3.2. Selection and Characteristics of Studies

**Figure 1:**
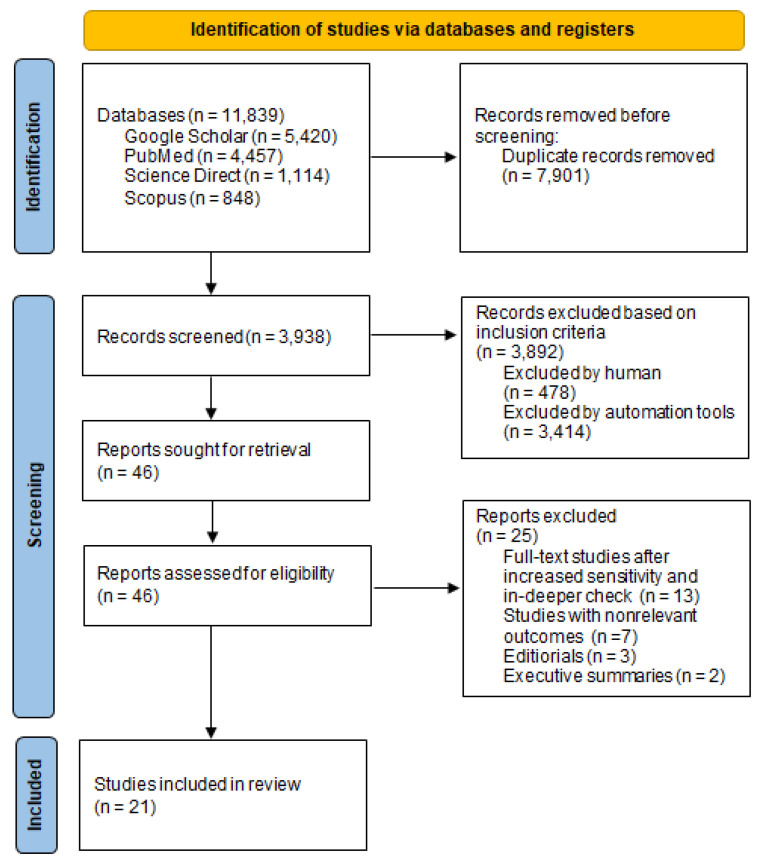
Collecting adequate studies on the basis of pre-defined criteria (PRISMA flow chart).

A search of electronic databases and scanning the reference lists yielded 11,839 studies. After removing duplicates, a total of 3938 studies were screened. Additional 3892 studies were excluded based on inclusion criteria and a total of 46 studies were screened and selected for eligibility. After increased sensitivity and in-deeper check, 25 studies with nonrelevant outcomes, editorials, and executive summaries were additionally excluded. Lastly, a total of 21 full-text studies were included in the systematic review (Figure 1).

Table 2 and Table 3 show in more detail the studies that met the set conditions and entered the qualitative analysis.

There were a total of 7675 participants. The highest number was 2324 [48], the lowest was 20 [43], while for only one study there were no data about the total number of participants [32]. The sample of participants was only female in five studies [34,38,40,41,43], while only one study had male participants only [47]. There were eight studies where the sample of participants was from the Faculty of Sport and Physical Education students [30,31,33,35,37,40,41,43], only one study had Criminal Police Academy students [36], while the rest of the studies had participants from different universities and faculties [42,43,44,45,46,47,48,49,50].

For measuring physical fitness, it should be mentioned Criminal Police Academy students [36], who were using standard battery test, prescribed by plan and program “Special physical education”. Two studies had students from Poland National University [45,46], one had Tsingua University students from China [44], and all three mentioned were using their own standardized battery test. Aqua-Pilates program [40], Bamboo dance program [39], hiking activities [35], and fitness yoga [38] were also evaluated and there were also two studies that conducted VO2max evaluation on the treadmill [49] and cycle ergometer [50]. The rest of the studies were using standard and already known physical fitness tests. There were evaluations of physical fitness based on practicing basketball games [32] and rhythmic gymnastics, both conducted on faculty [37], and concurrent power training with high-intensity interval cycling [43].

Two types of relationships were conducted. The first was the relationship between physical fitness and academic performance among Chinese University students [48] and the second one [50] was a relationship between depression, daily physical activity, physical fitness, and daytime sleep time in Japanese University students. In addition, only one study [49] was determining predictors of metabolic syndrome (eating speed, physical activity and cardiorespiratory fitness) in Korean University students.

Evaluation of physical activity was various. International Physical Activity Questionnaire (IPAQ) was used in four studies [42,44,47,48] and among those mentioned, only Osipov et al. [47] were using fitness center and sport school documents as evaluation of the physical activity. The self-assessment questionnaire was conducted only by Kaminska et al. [31], Kang et al. [49] had a regular questionnaire, while Shimamoto et al. [50] was the only study that monitored and evaluated physical activity using the accelerometer. Additionally, the mentioned questionnaires are valid and reliable [51,52,53], while the details of the accelerometer measurement have already been described in previous studies [54,55].

## 4. Discussion

The current study aimed to examine the scientific evidence regarding physical activity and physical fitness among university students. The universities are an ideal environment for the promotion of physical fitness and physical activity. The main findings of the current review are that university students show moderate levels of physical fitness and physical activity. However, when considering the results, it should be done with caution due to cultural differences, different faculties included in the review as well as the difference in educational systems. Moreover, some studies included students from the faculty of sports who have physical activities within the curriculum that must be taken into account. This was confirmed by Kaminska et al. [31] who found that students of the faculty of sports have better physical fitness compared to physiotherapy students, which was explained by a higher degree of physical activity in classes.

Additionally, when it comes to analyzing the impact of physical activities, such as aqua-Pilates [40], it was concluded that the group that implemented the program made statistically significant progress in terms of muscle strength, flexibility and balance. Meredith-Jones et al. [56] have also found similar results, that water exercises can lead to beneficial effects on cardiorespiratory fitness, strength and body fat. The weight loss in water removes the body load and helps in increasing the range of motion according to normal conditions, as well as significant energy expenditure [57]. In addition, such results are a consequence of the application of exercises that develop the mentioned abilities, because as already mentioned, the application of physical activities can increase the already acquired level of fitness and physical abilities. Fitness is an activity that consists of exercises that require the engagement of the whole body and develop both coordination and strength, as well as flexibility and balance [38].

Jourkesh et al. [30] have found that females performed better in flexibility tests than males, which is in full accordance with Ortega et al. [58]. Hiking activities conducted by Citozi et al. [35] found gender differences in balance tests, which is lined with other findings [59,60]. In accordance to previously mentioned studies, Zou et al. [39] have examined the influence of Bamboo dance and at the end of the program, the experimental group showed significant progress in balance (3.6%), agility (0.18%), strength (0.33%) and explosive power (0.42%). As the authors have considered low physical activity in university students, Bamboo dance interventions are needed. According to all mentioned facts, the main focus should be placed on reducing barriers that students experience that may impact their physical activity [61].

Wang [44] results showed that students with a higher degree of physical activity have 2.39 times better results in the strength test and 1.39 times in the long jump test. Osipov et al. [47] also used a standardized questionnaire to assess physical activity to divide participants into groups, and proved that participants with a higher degree of physical activity had better physical fitness compared to those with a lower degree. The results were even compared with the obtained results of some other countries, where Russian students show significantly better results compared to students from African countries [62,63], Turkey [64], Iran [65] and Ukraine [66], as well as several more European countries [67,68]. Two similar studies conducted by Griban et al. [45,46] gave two new answers. The first indicates the problem of increasing the level of fitness in higher institutions and the second is consisted of analyzing the dynamics of fitness on the same sample. Likewise, Adriana et al. [32] found that younger students have poorer results in physical fitness compared to older ones, in exact same variables. Suri et al. [41] are suggesting that there is a strong need for more active physical education programs that are appropriate for developing fitness and improving the health status of college-going students. Although the current state of physical fitness of these students was at a very unsatisfactory level, the authors emphasize that the system primarily does not provide the required level of physical fitness and work ability, which is why there is a great need to identify an adequate and efficient program that will improve the traditional physical education system. Kang et al. [49] found physical inactivity and poor cardiorespiratory function to metabolic equivalent of task risk. Students who are busy with their studies, part-time jobs and extracurricular sport clubs–physical activities that are normally considered to be beneficial [69,70] may cause excessive negative side effects [50]. In accordance with the current findings, similar results have been found in Western [71,72,73,74] and Asian countries [75,76,77].

Unlike all other studies in which the authors wanted to show that applying physical activity has a positive impact on the physical fitness of students, Mitrović et al. [36] decided to examine the impact of the absence of special physical education on physical fitness in an 8-month period. They came to the conclusion that the absence of this type of physical activity in students causes a significant decline in physical fitness, which may be an indication that already acquired physical fitness may decline if physical activity is not practiced. If there is more physical activity, the more physically fit individuals at the adequate level are there [78]. According to Rossomanno et al. [79], if we want to secure that these students could perform their duties, it is necessary to apply the monitored program for the development of physical fitness and physical activity during the whole year.

The main limitation of this study lies in cultural differences, where we included a wide range of universities, as well as differences in educational systems. As studies about physical activity interventions have rarely been enforced in some parts of included studies, further research should promote regular exercise in the university students. Despite the mentioned limitations, this study should be an important contribution to physical activity, as well as physical fitness research, and be beneficial to elucidating the primary negative factors.

## 5. Conclusions

Based on the obtained results, university students show a satisfactory level of physical activity and physical fitness. However, the results vary due to the different factors involved, which are mostly related to cultural differences and educational systems in different countries. The results for the students of the faculty of sport showed that physical activity has a positive effect on the development and maintenance of physical fitness and activity. As far as students from different faculties, this study suggests that further investigations should be conducted to promote daily exercise, as it may be beneficial for physical fitness and activity related to students health. 

## Figures and Tables

**Table 1:** PEDro scale results.

	Criterion											
Study	1	2	3	4	5	6	7	8	9	10	11	∑
Jourkesh et al. (2011) [30]	Y	N	Y	N	Y	N	N	Y	Y	Y	Y	6
Kaminska et al. (2012) [31]	Y	N	Y	N	Y	N	N	Y	Y	Y	Y	6
Adriana et al. (2012) [32]	Y	N	Y	Y	Y	N	N	Y	Y	Y	Y	7
Ribeiro et al. (2013) [33]	Y	N	Y	N	Y	Y	N	Y	Y	Y	Y	7
Elamaran (2014) [34]	Y	Y	Y	N	Y	N	N	Y	Y	Y	Y	7
Çitozi et al. (2016) [35]	Y	N	Y	Y	N	N	N	Y	Y	Y	Y	6
Mitrović et al. (2016) [36]	Y	N	Y	Y	Y	N	N	Y	Y	Y	Y	7
Moskovljević (2016) [37]	Y	N	Y	Y	Y	N	N	Y	Y	Y	Y	7
Skurikhina et al. (2016) [38]	Y	N	N	Y	Y	N	N	Y	Y	Y	Y	6
Zou et al. (2016) [39]	Y	Y	Y	Y	Y	Y	N	Y	Y	Y	Y	9
Özcan et al. (2018) [40]	Y	Y	Y	Y	Y	Y	N	Y	Y	Y	Y	9
Suri et al. (2018) [41]	Y	N	N	Y	N	N	N	Y	Y	Y	Y	5
Pituk (2019) [42]	Y	N	N	Y	Y	N	N	Y	Y	Y	Y	6
Spiliopoulou et al. (2019) [43]	Y	N	N	Y	N	N	N	Y	Y	Y	Y	5
Wang et al. (2019) [44]	Y	N	Y	N	Y	N	N	Y	Y	Y	Y	6
Griban et al. (2020) [45]	Y	N	Y	N	Y	Y	N	Y	Y	Y	Y	7
Griban et al. (2020) [46]	Y	N	Y	N	Y	Y	N	Y	Y	Y	Y	7
Osipov et al. (2020) [47]	Y	N	N	N	Y	Y	N	Y	Y	Y	Y	6
Zhai et al. (2020) [48]	Y	N	N	N	Y	Y	N	Y	Y	Y	Y	6
Kang et al. (2021) [49]	Y	N	N	N	Y	Y	N	Y	Y	Y	Y	6
Shimamoto et al. (2021) [50]	Y	N	Y	N	Y	Y	N	N	Y	Y	Y	6

Legend: 1—eligibility criteria; 2—random allocation; 3—concealed allocation; 4—baseline comparability; 5—blind subject; 6—blind clinician; 7—blind assessor; 8—adequate follow-up; 9—intention-to-treat analysis; 10—between-group analysis; 11 —point estimates and variability; Y—criterion is satisfied; N—criterion is not satisfied; ∑—total awarded points.

**Table 2:** Review of studies.

First Author and Year of Publication	Sample of Participants			PF	Results
	Number	Age (Years)	Groups		
Jourkesh et al. (2011) [30]	N-450 M-250 F-200	M-22.5 ± 8.25 F-22.75 ± 6.20	FSPE	SaR, CMJ, 10 m SR, Flex, Pu	M > F (CMJ, 10 m SR, Flex, Pu) F > M (SaR)
Kaminska et al. (2012) [31]	N-82 M-28 F-54	19–23	FSPE, PH	Su, dyn, SB	** FSPE and PH
Adriana et al. (2012) [32]	X	X	(BuchUni) EG, CG, BB	30 m sprint, Su, Pu, CMJ	EG results are below average
Ribeiro et al. (2013) [33]	N-257 M-125 F-132	M-20.4 ± 2.3 F-19.9 ± 1.91	FSPE	MAT, SaR, 12 min run-walk test	High-level of PF Low level of CRP
Elamaran (2014) [34]	F-45	18–20	(IndUniP) ImT, ItT, CG	Sq, Pu, HJ, bdps, pnk	* ImT and ItT
Çitozi et al. (2016) [35]	N-24 M-14 F-10	19–21	FSPE	SB	SB increased by 16.08% (open eyes) and 20.62% (closed eyes)
Mitrović et al. (2016) [36]	N-137 M-67 F-70	19–21	KPA	dhg, mif (dl), LJ, HJ, Su, Plps, Pu	F (dhg = 6.99%, LJ = 4.59%, HJ = 7.3%) M (LJ = 2.83%, HJ = 4.27%)
Moskovljević (2016) [37]	N-58 M-29 F-29	20–21	FSPE	SB, Flex, Els, Rls	High level of PF students who have passed RG
Skurikhina et al. (2016) [38]	F-40	19–24	2 students groups	Pu, MAT, Rls	** PF
Zou et al. (2016) [39]	N-30 M-18 F-12	20.8 ± 2.07	(ChiUni) EG (Bd), CG	Ag, Bd, Els, LJ	** EG
Özcan et al. (2018) [40]	F-60	18–25	(FSPE) EG (AP), CG	SaR, dhg, dynb, dynl, DB, VC	** EG (AP)
Suri et al. (2018) [41]	F-96	19	FSPE	SaR, Bst	PEC ** Flex
Pituk (2019) [42]	N-392 M-167 F-225	M-18.4 ± 0.74 F-18.4 ± 1	FilUni	20 m SR, SaR, Z, Cu, CMJ	F > M (SaR) M > F (20 m SR, CMJ, Cu)
Spiliopoulou et al. (2019) [43]	F-20	21.8 ± 2.8	FSPE (ST, STIAT)	CMJ	STIAT showed better results in CMJ
Wang (2019) [44]	N-1414	18–24	TsgUni	LJ, VC, IFff-DA	Higher PA degree students have 2.39× better scores at IFff-DA and 1.39× at LJ
Griban et al. (2020) [45]	N-369 M-195 F-174	19–24	PolNatUni, 16 groups	100, 2000 and 3000 m sprint, LJ, Plps, Pu, MAT, 4 × 9 SR, SaR	Unsatisfactory level of PF
Griban et al. (2020) [46]	N-394 M-199 F-195	19–24	PolNatUni, 16 groups	100, 2000 and 3000 m sprint, LJ, Plps, Pu, MAT, 4 × 9 SR, SaR	2nd year students show the best PF
Osipov et al. (2020) [47]	M-205 E1-127 E2-78	19–20	RusUniH, RusUniT	1mile-run test, Pu, sq, pnk	* of students who have the evidence of PA
Zhai et al. (2020) [48]	N-2324 M-1621 F-708	19.6 ± 0.6	3 ChiUni	VC, 50 m sprint, SaR, LJ, Su, Plps 1000 and 500 m run	PF ++ Agr no matter of LS
Kang et al. (2021) [49]	N-1183 M-832 F-351	23.2 ± 2.6	KorUni	VO2max on T	M > F VO2max
Shimamoto et al. (2021) [50]	N-95 M-52 F-33	18.9 ± 1.4	JapUni	VO2max on CE	VO2max = 42.5 ± 6.5 Ml/kg/min

Legend: N—total number of participants; M—male, F—female; X—no data; EG—experimental group; CG—control group; PF—physical fitness tests; PA—physical activity; PEC—physical education classes; CMJ—countermovement jump; CRP—cardiorespiratory fitness; Els—explosive legs strength; Rls—repetitive legs strength; DB—dynamic balance; SB—static balance; mif—maximal isometric force; sq—squat; dhg—dynamometer hand grip; dynb—dynamometry back muscles; dynl—dynamometry legs; dl—deadlift; bdps—bench dips; KPA—Criminal Police Academy students; TsgUni—Tsinghua University students; PolNatUni—Poliand National University students; FilUni—Filipino University students; SPE—special physical education; ImT—isometric training; ItT—isotonic training; CRT—cardiorespiratory fitness; VO2max—maximal oxygen uptake; T—treadmill; CE—cycle ergometer; PH—physiotherapy students; FSPE—faculty of sport and physical education students; JapUni—Japanese University students; KorUni—Korean Unversity students; ChiUni—Chinese University students; RusUniH—Russian University students of humanities specialties; RusUniT—Russian University students of technical specialties; BuchUni—Bucharest University; IndUniP—Indian University Polytechnic College; ST—strength training; STIAT—strength training and intensive aerobic training; Flex—flexibility; Pu—push-ups; Plps—pull-ups; Su—sit-ups; LJ—long jump; Z—zipper test; Cu—curl up test; HJ—high jump; IFff—isometric force of the finger flexor; DA—dominant arm; RG—rhytmic gymnastics classes; AP—aqua-pilates; sq—squat; pnk—plank; Agr—academic grades; MAT—modified abdomen test; SR—shuttle run; SaR—sit and reach; Bst—back scratch test; Bd—Bamby dance; Ag—agility; BB—basketball; VC—vital capacity; LS—lifestyle; * significant improvement; ** significant difference between groups; ++ positive correlation.

**Table 3:** Physical activity evaluation.

First Author and Year of Publication	Sample of Participants			PA Evaluation	Results
	Number	Age (Years)	Groups		
Kaminska et al. (2012) [31]	N-82 M-28 F-54	19–23	FSPE, PH	SAQ	Low level (M-10.7%, F-48.1%) Moderate level (M-35.7%, F-40.7%) High level (M-17.9%, F-5.6%) Vigorous level (M-35.7%, F-5.6%)
Pituk et al. (2019) [42]	N-392 M-167 F-225	M-18.4 ± 0.74 F-18.4 ± 1	FilUni	IPAQ	High level 37% Moderate level 48% Low level 15%
Wang et al. (2019) [44]	N-1414	18–24	TsgUni	CL-IPAQ	TA-41.50% LA-17.36% DA-7.5%
Osipov et al. (2020) [47]	M-205 E1-127 E2-78	19–20	RusUniH, RusUniT	FC, SS, IPAQ	* E1 in workplace PA * E2 in total PA
Zhai et al. (2020) [48]	N-2324 M-1621 F-703	19.6 ± 0.6	3 ChiUni	CS-IPAQ	** M in MVPA ** F in GPA
Kang et al. (2021) [49]	N-1183 M-832 F-351	23.2 ± 2.6	KorUni	Q	METs were gradual in order of PiA, FE and poor CRF
Shimamoto et al. (2021) [50]	N-95 M-52 F-33	18.9 ± 1.4	JapUni	Acc	EEPA and Ds were higher in part-time job students

Legend: N—total number of participants; M—male; F—female; CT—circuit training; A—aerobic; PH—physiotherapy students; FSPE—faculty of sport and physical education students; FilUni—Filipino University students; RusUniH—Russian University students of humanities specialties; RusUniT—Russian University students of technical specialties; ChiUni—Chinese University students; KorUni—Korean University students; JapUni—Japanese University students; TsgUni—Tsinghua University students; LA—learning activity; TA—traffic activity; DA—domestic work; IPAQ—International Physical Activity Questionnaire; SAQ—Self-Assessment Questionnaire; CL-IPAQ—Chinese long format of IPAQ; PA—physical activity; GPA—average grade point; MVPA—moderate-vigorous intensity physical activity; CS-IPAQ—Chinese short format of IPAQ; Q—questionnaire, BSIQ—Body Self Image Questionnaire; FC—fitness center documents; SS—sport school documents; PiA—physical inactivity; FE—fast eating; CRF—cardiorespiratory fitness; Acc—accelerometer; METs—metabolic equivalent; EEPA—energy exposure originating from physical activity per day; Ds—daily steps; * significant improvement; ** significant difference between groups.

## Data Availability

Not applicable.
